# Dose-Dependent Anti-Inflammatory and Neuroprotective Effects of an ****ανβ****3 Integrin-Binding Peptide

**DOI:** 10.1155/2013/268486

**Published:** 2013-11-17

**Authors:** Shu Han, Fan Zhang, Zhiying Hu, Yayi Sun, Jing Yang, Henry Davies, David T. W. Yew, Marong Fang

**Affiliations:** ^1^Institute of Neuroscience, Zhejiang University School of Medicine, 866 Yuhangtang Road, Hangzhou 310058, China; ^2^Department of Obstetrics and Gynecology, Hangzhou Red Cross Hospital, Hangzhou 310003, China; ^3^Zhejiang University School of Medicine, Hangzhou 310058, China; ^4^Brain Research Center, Faculty of Medicine, The Chinese University of Hong Kong, Hong Kong

## Abstract

Previous studies have shown that prevention of leukocyte infiltration by targeting integrins involved in transendothelial migration may suppress the clinical and pathological features of neuroinflammatory disease. This study was designed to investigate the effects of C16, an **α** **ν** **β**3 integrin-binding peptide, in an acute experimental allergic encephalomyelitis (EAE) rat model. Multiple histological and immunohistochemical staining, electron microscopy observation, ELISA assay, Western blot, and magnetic resonance imaging (MRI) were employed to assess the degree of inflammation, axonal loss, neuronal apoptosis, white matter demyelination, and extent of gliosis in the brain and spinal cord of differently treated EAE models. The results showed that C16 treatment could inhibit extensive leukocyte and macrophage accumulation and infiltration and reduce cytokine tumor necrosis factor-**α** (TNF-**α**) and interferon-**γ** (IFN-**γ**) expression levels. A significantly lower clinical score at the peak time of disease was also demonstrated in the C16 treated group. Moreover, astrogliosis, demyelination, neuronal death, and axonal loss were all alleviated in C16 treated EAE animals, which may be attributed to the improvement of microenvironment. The data suggests that C16 peptide may act as a protective agent by attenuating inflammatory progression and thus affecting the expression of some proinflammatory cytokines during neuroinflammatory disease.

## 1. Introduction

Multiple Sclerosis (MS) is a progressive autoimmune disease that invokes an inflammatory attack on the central nervous system (CNS) resulting in an accumulating disability [1, 2]. Experimental allergic encephalomyelitis (EAE) is a primary animal model of MS which is widely used for the evaluation of different drugs in MS treatment [3]. EAE includes the breakdown of the blood-brain barrier, infiltration of the CNS by CD^4^ T cells and macrophages, and activation of microglia and astrocytes. This results in inflammation followed by demyelination [1, 4, 5]. Activated microglia and astrocytes have been implicated in the secretion of a number of proinflammatory mediators, such as TNF-*α*, IFN-*γ*, and metalloproteinases, which act as inflammatory mediators and tissue damaging agents in the onset of EAE [1, 6, 7]. The remission phase of EAE is accompanied by increased production of immunoregulatory cytokine transforming growth factor-*β* (TGF-*β*) [8, 9]. As the disease progresses, defective remyelination due to the loss of oligodendrocytes and axonal degeneration can lead to the increase of clinical handicaps [10]. In addition to that, secondary neuronal loss and astrogliosis underlie the deterioration of EAE [11, 12].

 Leukocytes leave the circulation by crossing the vascular endothelium. The dynamic process of transendothelial migration includes the initial tethering of leukocytes to the vessel wall, followed by the rolling of these cells along the endothelium, forming tight adhesion to the endothelial surface, and ultimately moving through the intercellular junctions into the underlying tissue [13, 14]. Integrins are the molecular limbs of a cell, enabling the trafficking and entry of pathogenic leukocytes into inflamed tissues. *ανβ*3 is the most promiscuous member of the integrin family, which allows endothelial cells to interact with a wide variety of extracellular matrix components [13–16]. It is preferentially expressed on angiogenic blood vessels and supposed to have the function of improving angiogenesis [13–16]. Previous studies have also revealed that occupancy of the *ανβ*3 integrin could decrease monocyte binding to intercellular adhesion molecule-1 (ICAM-1) and block the process of monocytes migration across the endothelium [14, 15]. The synthetic C16 peptide (KAFDITYVRLKF), representing a functional laminin domain, may selectively bind to *ανβ*3 integrin, interfering with a leukocyte ligand required for transmigration and attenuating monocytes transmigration across endothelial cell layer *in vitro *[15, 16]. It also alleviates monocytes extravasation and macrophages activation in spinal cord contusion models *in vivo *[16, 17]. Most importantly, a previous study has shown that the C16 peptide does not affect systemic leukocyte counts and is not an immune-suppressant [16].

In MS, large numbers of leukocytes infiltrate through the blood-brain barrier into the CNS resulting in widespread tissue damage without any apparent infection [18–21]. Previous researches have suggested that infiltration of such a large number of leukocytes plays a central role in the development and progression of MS and EAE [18, 22–25]. Therefore, targeting neuroinflammatory reaction has been an important remedial point to alleviate the pathological features and clinical motor symptoms. Since C16 has been shown to exert anti-inflammatory activity in traumatic models of CNS [16], we hypothesize that intravenous administration of C16 would also limit inflammatory cells infiltration in EAE models. In order to optimize the application dose, a C16 dose response study was carried out and the most appropriate therapeutic time window was discussed.

## 2. Materials and Methods

### 2.1. Animals and EAE Induction

 A total of 100 adult male Lewis rats were obtained from Zhejiang University Laboratory Animal Services Centre. Of these, 4 were taken as normal control and the remaining 96 were randomly assigned into two vehicle control groups (*n* = 32, 16/group) and four C16 treatment groups (*n* = 64, 16/group). Experiments were carried out in accordance with NIH Guidelines for the Care and Use of Laboratory Animals, with approval from the Animal Ethics Committee at Zhejiang University.

EAE was induced in the rats for C16 treatment and the vehicle control groups by subcutaneously injection of 0.2 mL 1 : 1 mixture of guinea pig spinal cord homogenate (GPSCH) and complete Freud adjuvant (CFA), containing 0.5 mg of heat killed *mycobacterium tuberculosis* (Difco Laboratories, Detroit, MI). The rats in the normal control group were injected with CFA emulsified 1 : 1 with 0.9% saline [5, 20]. Immediately after CFA injection, and again after 48 hours, the rats received intraperitoneal injection of 300 ng Pertussis toxin (Sigma) twice. Beginning on day 7, the animals were weighed and assessed for clinical signs of disease on a daily basis. Disease severity was assessed using a scale ranging from 0 to 5: grade 0 = no signs, grade 1 = partial loss of tail tonicity, grade 2 = total loss of tail tonicity, grade 3 = unsteady gait and mild paralysis, grade 4 = hind limb paralysis and incontinence, and grade 5 = moribund or death [21]. The EAE model was generally considered a success if its score exceeded 2, and scoring was continuallyd performed by people blinded to the treatments of the animals until the time of sacrifice.

### 2.2. Intravenous Injection of C16

C16 peptide was dissolved in distilled water with 0.3% acetic acid. The peptide solution was sterilized through a 0.22 *μ*m disc filter and neutralized to pH 7.4 with NaOH. This solution was buffered by adding an equal volume of sterile phosphate-buffered saline and the final concentration was 2 mg/mL. The vehicle solution was prepared in the same manner without adding the peptide.

For the dose dependent test, the C16 application rats were divided into low (0.5 mg/per day, *n* = 16), medium (1 mg/day, *n* = 16), and high (2 mg/day, *n* = 16) treatment groups; each received 1 mL of C16 solution of different concentrations. The control (vehicle) group (*n* = 16) was treated with 1 mL of vehicle solution, via intravenous injection of the tail vein. The first dose was given immediately after receiving EAE induction (following the GPSCH injection and Pertussis toxin injection); thereafter, the solutions were injected intravenously each day for a period of two weeks.

In order to establish the appropriate therapeutic time window, another 32 rats were assigned into a vehicle and a C16 late treated group (16/group). In the latter group, the first dose of C16 (1 mg/day) was given 10 days after immunization, when the clinical handicaps of motor function began to emerge.

### 2.3. Perfusion and Tissue Processing

Animals of vehicle control and C16 treatment groups were sacrificed at 2 weeks and 8 weeks (5/time point in each group) after immunization. Rats were anesthetized with sodium pentobarbital, and then after receiving an *in vivo* MRI scan, they were perfused intracardially with cold saline followed by 4% paraformaldehyde in 0.1 M phosphate buffer (pH 7.4). The spinal cord and brain tissues were carefully harvested and dissected. 1 cm of the lumbar spinal cord and half of each animal's brain were fixed in the same fixative for 4 h and then transferred into 30% sucrose in PBS until the tissue sank to the bottom of the container. Twenty *μ*m thick sections were cut on freezing microtome through the coronal plane of the brain and the transverse plane of spinal cord using a Leica cryostat and then mounted onto 0.02% poly-L-lysine-coated slides. All sections were collected for histological assessment and immunohistological and immunofluorescent staining. The remains of the central nervous tissue were fixed in 2.5% glutaraldehyde solution and then examined by transmission electron microscope [15, 23].

### 2.4. MR Imaging

MR scanning was performed using a clinical 3.0T MRI machine (Signa HDxt 3T) equipped with a dedicated solenoid rat coil. Rats were anaesthetized and placed in the cradle supine. T2-weighted sequences (15 contiguous coronal slices of 1.5 mm) were collected with the following characteristics: TE = 120 ms, TR = 3200 ms, slice thickness = 1.5 mm, slice = 15, 320 × 192 matrix, field of view = 60 × 60 mm, flip angle = 90° [26].

### 2.5. Histology Assessment

Hematoxylin and eosin (H&E) staining and cresyl violet (Nissl) staining were employed to assess inflammation and neuron survival, respectively. Digital images were collected using a Nikon TE-300 microscope in 3 vision fields/section with ×200 magnification under a bright field. Neuron counts from both spinal cord anterior horns were performed and restricted to the neurons with well-defined nuclei as well as a cell body with adequate amount of endoplasmic reticulum. An assessment of the severity of inflammatory cell infiltration was done by a conventional H&E staining and scaled as follows [22]: 0, no inflammation; 1, cellular infiltrates only around blood vessel and meninges; 2, mild cellular infiltrates in parenchyma (1−10/section); 3, moderate cellular infiltrates in parenchyma (11−100/section); and 4, serious cellular infiltrates in parenchyma (100/section). 

Luxol fast blue (LFB) staining was used to evaluate the degree of axon demyelination, as previously described [15]. Demyelination was scored as follows using the following scale [1]: 0, normal white matter; 1, rare foci; 2, a few areas of demyelination; 3, confluent perivascular or subpial demyelination; 4, massive perivascular and subpial demyelination involving one half of the spinal cord with presence of cellular infiltrates in the CNS parenchyma; and 5, extensive perivascular and subpial demyelination involving the whole cord section with presence of cellular infiltrates in the CNS parenchyma.

Bielschowsky silver staining was performed as described previously to estimate axonal loss [23]. Axonal loss was assessed using the following scale [1]: 0, no axonal loss; 1, a few foci of superficial axonal loss involving less than 25% of the tissues; 2, foci of deep axonal loss that encompassed over 25% of the tissue; and 3, diffused and widespread axonal loss.

### 2.6. Immunohistochemical Staining

 A ring of wax was applied around the sections with a PAP pen (Invitrogen, Carlsbad, CA, USA). After rinsing in 0.01 M Tris-buffered saline (TBS) for 10 min, the sections were permeabilized and blocked with 0.3% Triton X-100/10% normal goat serum in 0.01 M PBS for 30 min and then incubated with polyclonal rabbit antibodies: anti-CD4 (1 : 500, AbCam, Cambridge, MA), anti-tumor-necrosis-factor alpha (TNF-*α* 1 : 1000, ProSci Incorporated, CA, USA), anti-caspase-3 (1 : 500; Cayman Chemical, Ann Arbor, MI), and mouse antimyelin basic protein (MBP, 1 : 500, AbCam, Cambridge, MA) overnight at 4°C. The process of immunohistochemical staining was performed as described previously [23]; primary antibody omission controls were used in order to confirm further the specificity of the immunohistochemical labeling. Five sections from the motor cortex and anterior horns of spinal cord of each animal were randomly selected and images were photographed under ×200 magnification in 3 vision fields/section. The caspase-3 and TNF-*α* immunoreactive cells were counted, and the immunoreactive areas for CD4 and MBP were analyzed with NIH image software. 

### 2.7. Immunofluorescence Staining

The sections were pretreated with the same method described above and incubated with primary monoclonal mouse antibodies CD45 and CD68/ED1 (1 : 100; Santa Cruz Biotechnology, Santa Cruz, CA) and polyclonal rabbit anti-glial fibrillary acidic protein (GFAP 1 : 200, Thermo Fisher Scientific, Waltham, MA). The process of immunofluorescence staining was performed as described previously [23]; primary antibody omission controls were used in order to confirm further the specificity of the immunohistochemical labeling. Immunoreactive areas of GFAP, CD45, and CD68 were analyzed with NIH image software.

### 2.8. Processing for Electron Microscopy

Tissues fixed with 2.5% glutaraldehyde were washed 3 times with 0.1 M PB. Postfixed tissues were placed in 1% osmium tetroxide at 4°C overnight and then washed 3 times with 0.1 M PB. The processing for electron microscopy was performed as described previously [23]. Images were captured first at low resolution and then at higher magnification in different regions of the white matter.

### 2.9. Cytokine Quantification by Enzyme-Linked Immunosorbent Assay (ELISA)

Peripheral blood samples were collected from rats that had been sacrificed by decapitation at 2 weeks and 8 weeks after immunization (*n* = 3 per time point/each group). Plasma samples were collected on ice (centrifuged for 20 minutes at 1000 ×g within 30 minutes of collection, and then at 10,000 ×g for 10 minutes at 4°C for complete platelet removal) using heparin as an anticoagulant. All samples were aliquot and stored at −80°C. To assess cytokine expression, plasma samples were incubated in 96-well plates precoated with antibodies to IFN-*γ* (BioLegend Inc., San Diego, CA) and TGF-*β* (R&D Systems, Minneapolis, MN) at 37°C for 60 min. HRP-conjugated goat anti-rabbit IgG (Bio-Rad) diluted at 1 : 2,000 was used as the secondary antibody. Optical density was measured at 450 nm on Model 680 Microplate Reader (Bio-Rad Laboratories, Corston, UK). The optical density was quantified by GraphPad Prism 4 (GraphPad Software, Inc).

### 2.10. Western Blotting

The rats were sacrificed by decapitation at 2 weeks and 8 weeks after immunization (*n* = 3 per time point/each group), and from each animal, the whole brain cortex and a 10 mm lumbar spinal cord segment were prepared for Western blotting, which was performed as described previously [23]. Total proteins were extracted with 2 mM PMSF in 1 mL ice-cold RIPA buffer added protease inhibitor cocktail. SDS-PAGE was performed on 12% polyacrylamide slab gel, and separated proteins were electrophoretically transferred to PVDF membrane in a Bio-Rad TransBlot apparatus. After blocking nonspecific binding sites with bovine serum albumin, each membrane was incubated for 12 h at room temperature with primary rabbit polyclonal anti-GFAP (1 : 500), anti-TNF-*α* (1 : 2000), and anti-caspase-3 (1 : 500). To normalize protein bands to a gel loading control, membranes were washed in TBST and reprobed with rabbit anti-*β*-actin (1 : 5,000, AbCam, MA) followed by an incubation with peroxidase-conjugated goat anti-rabbit (1 : 5,000, Santa Cruz, CA) and ECL detection. For the negative control, the primary antibody was omitted.

### 2.11. Statistical Analysis

All statistical graphs were created in GraphPad Prism software version 4.0. Kruskal-Wallis nonparametric analysis was conducted and followed by a nonparametric Mann-Whitney test on each pair of data. Data were analyzed by SPSS 13.0 software, and *P* values less than 0.05 were considered statistically significant.

## 3. Results 

### 3.1. The C16 Attenuated Perivascular/Parenchymal Inflammation and Decreased the Size of the Lesion Site

**Figure 1:**
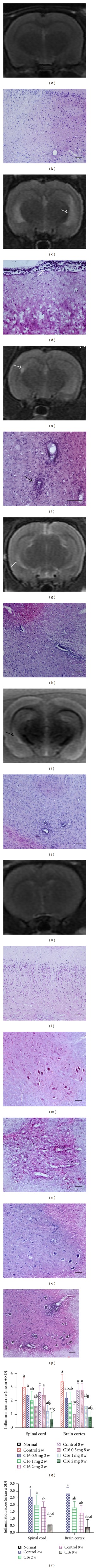
The appearance of lesion site in each group observed with MRI scanning was in accord with the severity of inflammatory cells infiltration showed by H&E staining: bar = 100 *μ*m. (a) Normal group, T_2_W image of brain, without detectable changes occuring in the white matter. (b) The same animal as in (a). Histological staining. (c) Vehicle control rats at week 2 after immunization, T_2_W image. An arrow indicates clear hyperintensity area in the white matter. (d) The same animal as in (c). H&E staining, an arrow shows abundant cellular infiltrates under meninges. (e) 0.5 mg/day C16 treated EAE rats at week 2 after immunization. T_2_W image, an arrow showed a focal area with increased intensity. (f) The same animal as in (e). H&E staining. The arrow denotes “perivascular cuffing” of inflammatory cells. (g) C16 late treated EAE rats at week 2 after immunization. T_2_W image, an increased intensity lesion area was defined by arrow. (h) The same animal as in (g). H&E staining. (i) At week 8 after immunization. T_2_W image, arrow denotes an increased intensity lesion area, the same animal as in (j). H&E staining showed less severe inflammatory cell infiltration, and a lesion area was found at week 8 smaller than that at week 2 after immunization. (k) 2 mg/day C16 treated EAE rats at week 8 after immunization, T_2_W image. (l) The same animal as in (k). H&E staining. ((m)–(p)) Diffuse infiltration of inflammatory cells was observed in the spinal cord of the vehicle control rats, and attenuated in C16 treated EAE rats at week 2 after immunization. H&E staining, traverse section through the lumbar spinal cord; bar = 100 *μ*m. Normal rats group (m), vehicle control rats (n), 2 mg /day C16 treated EAE rats (o), and C16 late treated EAE rats (p). (q) Medium and high dose C16 treatment reduced inflammatory cells showed by inflammation score. ^a^ *P* < 0.05 versus normal rats; ^b^ *P* < 0.05 versus vehicle control rats at week 2 postimmunization group; ^f^ *P* < 0.05 versus vehicle control rats; ^g^ *P* < 0.05 versus 0.5 mg/day C16 treated EAE rats at week 8 after immunization. (r) C16 late treatment also inhibited inflammatory cells infiltration to a certain extent showed by inflammation score. ^a^ *P* < 0.05 versus normal rats; ^b^ *P* < 0.05 versus vehicle control rats at week 2 after immunization; ^c^ *P* < 0.05 versus C16 treated EAE rats at week 2 postimmunization group; ^d^ *P* < 0.05 versus vehicle control rats at week 8 after immunization.

At the peak time of the disease (2 weeks after immunization), H&E staining of the cerebral cortex and spinal cord (Figure 1) revealed a significant increase of the cellular density in EAE rats treated with vehicle. Diffuse infiltration of inflammatory cells appeared in surrounding blood vessels and under the meninges; there was also wide infiltration of brain tissue parenchyma (Figure 1(d)). Clear lesion focus with hyperintensity was detectable in the white matter by MRI T_2_W scanning (Figure 1(c)). In the spinal cord, the gray matter showed a marked cellular infiltration which was not confined to the anterior or dorsal horns but extended throughout the gray matter and white matter (Figure 1(n)). After C16 treatment for 2 weeks at different doses, less severe perivascular and parenchymal infiltration of inflammatory cells (mixed with macrophages, microglia, and lymphocytes) within the spinal cord and brain tissue was observed (Figure 1). In general, when treated with a low dose of C16 (0.5 mg/day), the extent of inflammatory cells infiltration, the number of perivascular infiltration focus points, and the size of hyperintensity lesion sites in T_2_W images were significantly more abundant and larger in size than in the medium (1 mg/day) and in high (2 mg/day) dose C16 treated groups (Figures 1(e) and 1(f)). There were no significant differences in inflammatory cell infiltration between the medium and high dose groups (*P* > 0.05, Figure 1(q)). The C16 late treated group, which received C16 application 10 days after immunization, also presented a decrease of inflammation score compared with the vehicle control (*P* < 0.01, Figure 1(r)).

Eight weeks after immunization, visible lesion sites could still be detected by T_2_W scan (Figure 1(i)), but the extent of infiltration and the number of perivascular infiltration focus sites decreased compared with 2 weeks after immunization in the vehicle control (Figures 1(j)–1(l)). The low dose C16 treatment did not show evident amelioration of inflammation, but the medium and high dose treatments all exhibited remarkable improvement in suppressing the inflammatory cells infiltration (*P* < 0.001, Figure 1(q)). In the C16 late treated case, the escaping inflammatory cells also noticeably decreased compared with the vehicle control (*P* < 0.001, Figure 1(r)).

**Figure 2:**
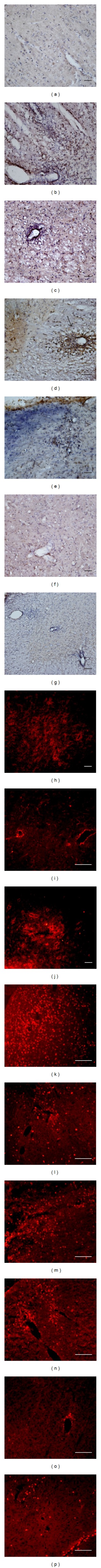
((a)–(g)) C16 treatment attenuated the CD4^+^ lymphocytes extravasation in CNS both at weeks 2 and 8 after immunization. CD4 immunostaining, counterstained with hematoxylin; bar = 100 *μ*m. At week 2 after immunization, normal group (a), vehicle control rats (b) (with severe cellular infiltration within parenchyma and surrounding blood vessel), 1 mg /day C16 treated EAE rats (c), C16 late treated EAE rats (d) (the arrow denotes “perivascular cuffing” of CD4^+^ lymphocyte). At week 8 after immunization, vehicle control rats (e), and 1 mg/per day C16 treated EAE rats (f), and C16 late treated EAE rats (g). ((h)–(j)) The C16 treatment attenuated the leukocytes infiltration in CNS both at weeks 2 and 8 after immunization. TRITC conjugated CD45 immunofluorescent staining. At week 2 after immunization, vehicle control rats (h), 2 mg/per day C16 treated EAE rats (i), and C16 late treated EAE rats (j). ((k)–(p)) The C16 treatment attenuated macrophages extravasation in CNS both at weeks 2 and 8 after immunization. TRITC conjugated CD68 immunofluorescent staining. At week 2 after immunization, vehicle control rats (k), 2 mg/per day C16 treated EAE rats (l), and C16 late treated EAE rats (m). At week 8 after immunization, vehicle control rats (n), 2 mg/day C16 treated EAE rats (o), and C16 late treated EAE rats (p).

**Figure 3:**
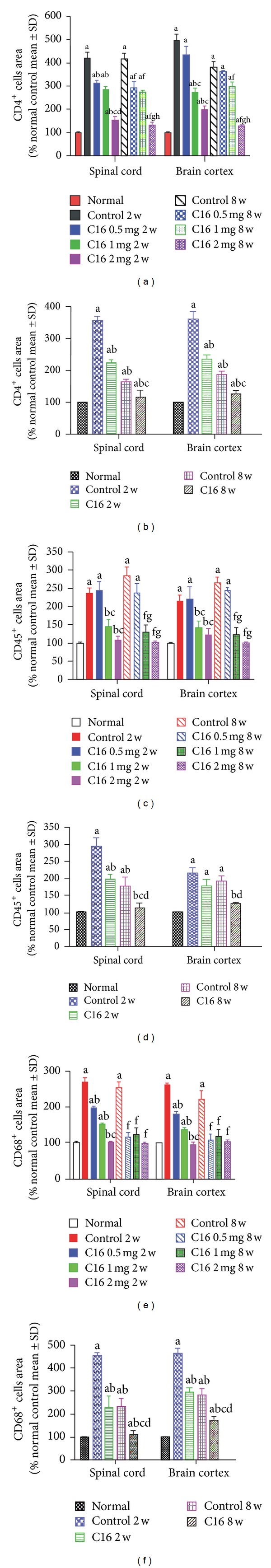
Medium- to high-dose C16 therapy reduced CD4^+^ lymphocytes extravasation (a). C16 late treatment also inhibited CD4^+^ lymphocytes extravasation to a certain extent (b). Medium to high-dose C16 treatment also reduced CD45^+^ leukocytes infiltration (c). C16 late treatment also inhibited CD45^+^ leukocytes infiltration to a certain extent (d). Medium to high-dose C16 treatment reduced CD68^+^ macrophages extravasation (e). Late treated C16 treatment also inhibited CD68^+^ macrophages extravasation to a certain extent (f). ((a), (c), (e)) ^a^ *P* < 0.05 versus normal rats; ^b^ *P* < 0.05 versus vehicle control rats at week 2 postimmunization group; ^c^ *P* < 0.05 versus 0.5 mg/per day C16 treated EAE rats at week 2 after immunization. ^f^ *P* < 0.05 versus vehicle control rats at week 8 after immunization; ^g^ *P* < 0.05 versus 0.5 mg/per day C16 treated EAE rats at week 8 after immunization. ((b), (d), and (f)) ^a^ *P* < 0.05 versus normal rats; ^b^ *P* < 0.05 versus vehicle control rats at week 2 postimmunization group; ^c^ *P* < 0.05 versus C16 treated EAE rats at week 2 postimmunization group; ^d^ *P* < 0.05 versus vehicle control rats at week 8 after immunization.

For determination of the types of inflammatory cells, the immunostaining of CD4 (a marker for extravasated T lymphocytes), CD45 (a pan-leukocyte marker for leukocytes), and CD68 (for activated microglia and extravasated macrophages) was done. The results showed that cells positive for CD4, CD68, and CD45 all increased remarkably in both grey and white matter of CNS in the vehicle control rats (Figure 2). An evident decline of T lymphocyte infiltration, leukocytes extravasation, and macrophages activation was detected in C16 treated group (Figures 2(c), 2(d), 2(f), 2(g), 2(i), 2(j), 2(l), 2(m), 2(o), and 2(p)), especially in the medium and high dose treated groups (*P* < 0.001, Figures 3(a), 3(c), and 3(e)). The C16 late treatment also reduced the CD4, CD68 and CD45 labeled inflammatory cells in both week 2 and 8 after immunization (*P* < 0.01, Figures 3(b), 3(d), and 3(f)).

### 3.2. C16 Treatment Inhibited Demyelination and Prevented Axon Loss

**Figure 4:**
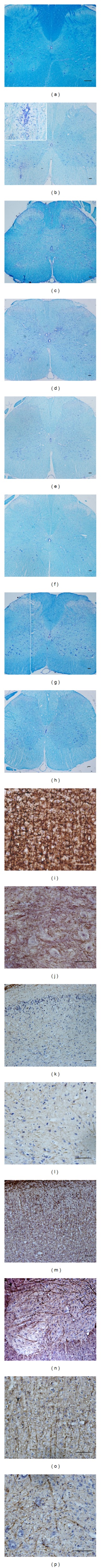
Luxol fast blue staining and MBP immunostaining for spinal cord and cerebral cortex. ((a)–(h)) C16 treatment prevented demyelination in spinal cord and cerebral cortex both at weeks 2 and 8 after immunization; Luxol fast blue staining; traverse section through the lumbar spinal cord; bar = 100 *μ*m. At week 2 after immunization, normal rats (a), vehicle control rats (b) (inserted image depicting the inflammatory cells infiltrated demyelination area in anterior funiculus), 2 mg/per day C16 treated EAE rats (c) and C16 late treated EAE rats (d). At week 8 after immunization, vehicle control rats (e), 0.5 mg (f) and 2 mg (g)/day C16 treated EAE rats, and C16 late treated EAE rats (h). ((i)–(p)) C16 treatment prevented demyelination in spinal cord and cerebral cortex both at weeks 2 and 8 after immunization, MBP immunostaining, counterstained with hematoxylin ((i), (k), (m), and (o) coronal sections of motor cortex; (j), (l), (n), and (p) traverse sections through the lumbar spinal cord). At week 2 after immunization, normal rats ((i), (j)), vehicle control rats ((k), (l)), 2 mg/per day C16 treated EAE rats ((m), (n)) and C16 late treated EAE rats ((o), (p)).

By Luxol fast blue (LFB) staining and MBP (one of the major central myelin proteins) immunohistochemical staining, we checked the total demyelination condition of each group. Massive perivascular and confluent demyelinated areas were present in the parenchyma of the CNS of vehicle control rats at the peak time of disease (week 2 after immunization) (Figures 4(b), 4(k), and 4(l)). At the same time point, when treated with different doses of C16 peptide, the visible areas of demyelination gradually declined (Figures 4(c), 4(d), 4(m), and 4(n)). In the high dose C16 treated group, only rare foci of demyelination could be found (Figures 4(c) and 4(g)). At week 8 after immunization, the demyelination condition in vehicle control was still obvious, while the myelin loss was significantly reduced by medium and high dose C16 treatment (Figures 4(e)–4(g)). Similarly, the late C16 treatments also remarkably reduced the demyelination score and improved the MBP labeled myelin area compared with vehicle control at the same time point (Figures 4(d), 4(h), 4(o), and 4(p); Tables 1 and 2). 

**Figure 5:**
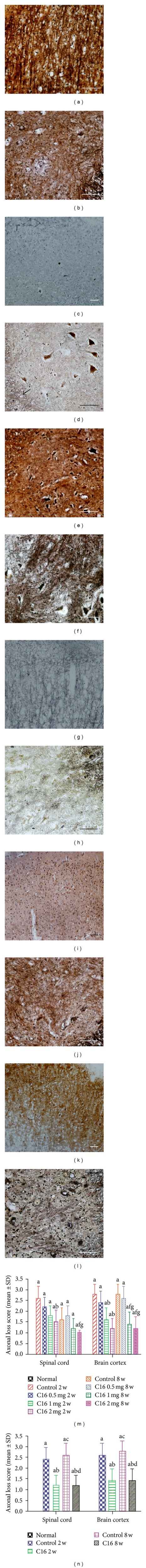
C16 treatment alleviated axonal loss in spinal cord and cerebral cortex revealed by Bielschowsky staining both at weeks 2 and 8 after immunization. ((a), (c), (g), (i), and (k) coronal sections of motor cortex; (b), (d), (e), (f), (h), and (j) traverse sections through the lumbar spinal cord); bar = 100 *μ*m. At week 2 after immunization, normal rats group ((a), (b)), vehicle control rats ((c) axons were undergoing gradual loss and exhibiting deformed and ovoid formation; (d) an arrow shows the axon loss in white matter); 2 mg/per day C16 treated EAE rats (e) and C16 late treated EAE rats (f). At week 8 after immunization, vehicle control rats ((g), (h)), 2 mg/per day C16 treated EAE rats ((i), (j)). C16 late treated EAE rats ((k), (l)). (m) Medium to high-dose C16 treatment prevented axon loss by an estimate of axonal loss score. ^a^ *P* < 0.05 versus normal rats; ^b^ *P* < 0.05 versus vehicle control rats at week 2 after immunization group; ^f^ *P* < 0.05 versus vehicle control rats at week 8 after immunization. ^g^ *P* < 0.05 versus 0.5 mg/per day C16 treated EAE rats at week 8 after immunization. (n) C16 late treatment prevented demyelination to a certain extent by axonal loss score. ^a^ *P* < 0.05 versus normal rats; ^b^ *P* < 0.05 versus vehicle control rats at week 2 postimmunization group; ^c^ *P* < 0.05 versus C16 treated EAE rats at week 2 postimmunization group; ^d^ *P* < 0.05 versus vehicle control rats at week 8 after immunization.

The vehicle control rats displayed severe axonal loss both in white and gray matters of CNS at week 2 after immunization; a similar condition existed in week 8 after immunization. The Bielschowsky staining impregnation revealed that the injured axons have swelling, with a deformed and ovoid formation (Figures 5(c) and 5(d)). Compared with vehicle control, there was no notable difference in axonal number in the low dose C16 treated group, but more axons with relatively normal formation were kept in medium and high dose treated EAE rats (Figures 5(e), 5(i), 5(j), and 5(m)). Even when the treatment was delayed to day 10 post immunization, medium dose C16 application still effectively suppressed the axonal loss score compared with the vehicle control of the same time point (Figures 5(f), 5(k), 5(l), and 5(n)). 

**Figure 6:**
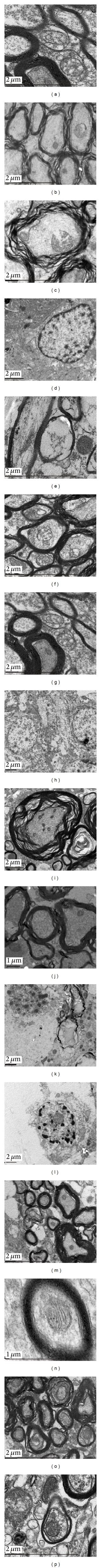
Electron micrograph demonstrated prevention of myelination or axons loss and inhibition of neuronal apoptosis in C16 treated rats. (a) Normal rats group (normal myelinated axons exhibited dark-rings-shaped myelin sheath surrounding axon). ((b)–(d)) Vehicle control rats at week 2 after immunization. A considerable amount of the myelin sheath displayed splitting, vacuolus, and loose and fused change, and the axon is shrunken and is dissolving ((b), (c)). The neuron showed apoptotic signs of a shrunken nucleus with condensed, fragmented, and marginated nuclear chromatin (d). At week 2 after immunization, 0.5 mg (e), 1 mg (f), and 2 mg ((g), (h))/per day C16 treated EAE rats. Some vacuolated and fused myelin sheaths were observed, but most of dark-rings-shaped myelin sheath structure has been kept ((f), (g)); the ultrastructure of neuron was still kept relatively normal (h). Visible myelin lamella cleavage, splitting, and fragmentation were detected in C16 late treated EAE rats (i). ((j)–(l)) Vehicle control rats at week 8 after immunization. Many myelin lamellae were still undergoing vesicular disintegration, but some were remyelinated; invasion of a myelin sheath by a macrophage containing myelin debris was illustrated in (k), and apoptotic neurons could be found in (l). ((m), (n)) 2 mg/per day C16 treated EAE rats at week 2 after immunization, (n) illustrating a rescued axons with a intact myelin. ((o), (p)) C16 late treated EAE rats at week 8 after immunization. More fibers were being remyelinated in these C16 treated animals, (p) showing newly formed myelin sheaths.

Transmission electron microscopy examination further revealed that a considerable amount of the myelin sheath displayed splitting and vacuoles changes in the vehicle control group (Figures 6(b) and 6(c)). Meanwhile, the axons were covered by disrupted myelin sheaths, and in some places, the axons even disappeared (Figure 6(c)). The neurons showed apoptotic signs of shrunken nuclei with condensed, fragmented, and marginated nuclear chromatin (Figure 6(d)). The low dose C16 treated EAE rats revealed similar disrupted myelin and shrunken axons (Figure 6(e)), but more lightly vacuolated myelin sheaths were found in medium and high dose C16 treated EAE rats and the corresponding axons and neighboring nuclei were close to the normal ultrastructure (Figures 6(f)–6(h)). However, more seriously loosened myelin appeared in the CNS of late C16 treated EAE rats compared with the early treated rats of the same dose (Figure 6(i)). At week 8 after immunization in the vehicle control group, some myelin lamellae were still undergoing vesicular disintegration (Figure 6(j)). The disrupted myelin sheaths had been invaded by macrophages containing myelin debris (Figure 6(k)), and the nucleus of neurons exhibited apoptotic signs (Figure 6(l)), and some were undergoing remyelination. In the meantime, more remyelinated fibers appeared both in early and late C16 treated EAE rats (Figures 6(m)–6(p)).

### 3.3. C16 Delayed the Disease Progression and Alleviated the Disease Severity

**Figure 7:**
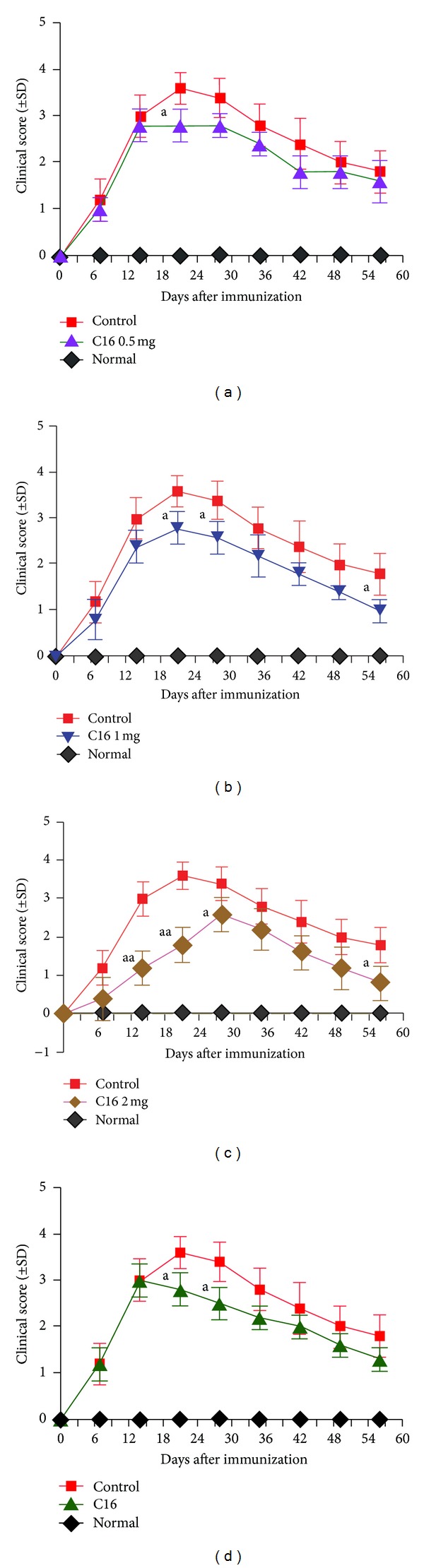
C16 treatment ameliorated the disease progression and alleviated the disease severity. ((a)–(c)) The clinical progression of EAE was attenuated after medium (b) and high (c) dose C16 applications. ^a^ *P* < 0.05 versus vehicle treated group at the same time point. ^aa^ *P* < 0.01 versus vehicle treated group at the same time point. (d) The clinical progression of EAE was attenuated by the C16 late treatment. ^a^ *P* < 0.05 versus vehicle treated group at the same time point.

In the vehicletreated rats, disease symptoms appeared on days 9-10 after immunization. The acute phase of the disease began with a sharp increase of motor symptoms (average clinical score: 3.5–4.0) which peaked at week 2 after immunization (Figure 7). Thereafter, the clinical score gradually declined. At week 8 after immunization, the clinical score of surviving vehicletreated animals returned to a level of 1.5-2 (Figures 7(a)–7(d)). Although animals treated with C16 showed a similar disease course to the vehicle control group, the low, medium, and high dose C16 treatments all could clearly suppress the clinical score in the peak stage (weeks 2–4 after immunization) and the medium and high dose treatments also accelerated the progress of functional recovery. With the increasing dosage, the amelioration of clinical signs was more conspicuous (*P* < 0.01 in high dose treatment, Figures 7(a)–7(c)). As for the late treated groups, there was no visible difference in clinical signs at the first two weeks after immunization, whereas the C16 treated group exhibited a significant decline in disease severity at weeks 3-4 after immunization (Figure 7(d)).

### 3.4. C16 Ameliorated Reactive Astrocytes Proliferation and Reactive Gliosis in the EAE Rat Model

**Figure 8:**
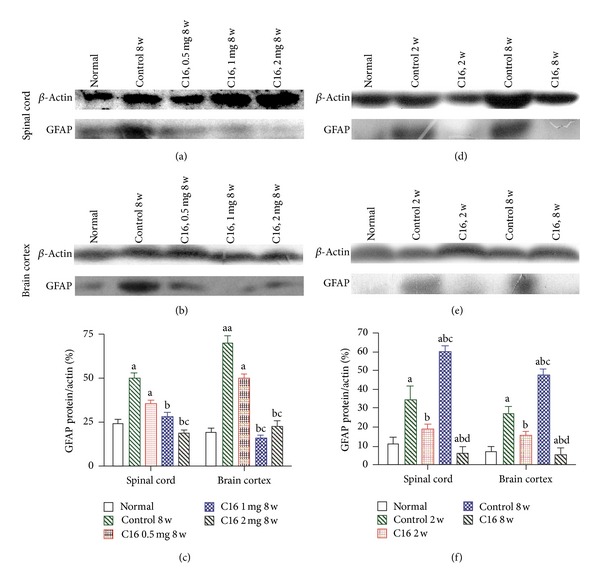
Medium and high-dose C16 treatment decreased evidently the expression levels of GFAP both in spinal cord (a) and cerebral cortex (b), showed by Western blotting analysis. (c) The levels of GFAP expression revealed by Western blotting analysis at week 8 after immunization: ^aa^ *P* < 0.01 versus normal rats, ^a^ *P* < 0.05 versus normal rats, ^b^ *P* < 0.05 versus vehicle control rats at week 8 postimmunization group, ^c^ *P* < 0.05 versus 0.5 mg/per day C16 treated EAE rats at week 8 after immunization. The levels of GFAP expression were declined remarkably by late C16 application both in spinal cord (d) and cerebral cortex (e) compared with the vehicle control. (f) The levels of GFAP expression revealed by Western blotting analysis: ^a^ *P* < 0.05 versus normal rats, ^b^ *P* < 0.05 versus vehicle control rats, ^c^ *P* < 0.05 versus C16 treated EAE rats at week 2 postimmunization group and ^d^ *P* < 0.05 versus vehicle control rats at week 8 after immunization.

**Figure 9:**
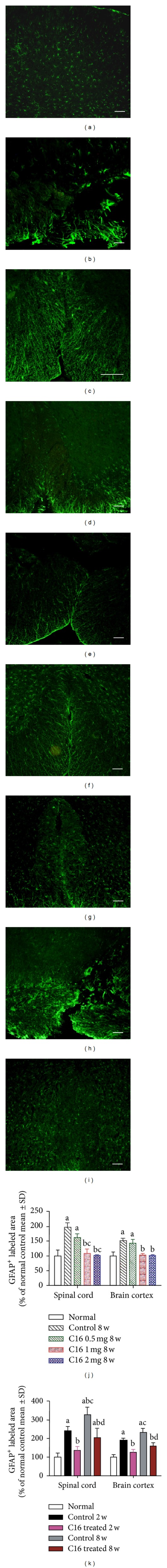
The C16 treatment inhibited reactive gliosis revealed by FITC-conjugated GFAP immunofluorescent staining; traverse section through the lumbar spinal cord; bar = 100 *μ*m. At week 8 after immunization, normal rats group (a), vehicle control rats (b), 0.5 mg (c), 1 mg/per day C16 treated EAE rats (d), and 2 mg/per day C16 treated EAE rats (e). Vehicle late treated EAE rats (f) and C16 late treated EAE rats (g) at week 2 after immunization. Vehicle late treated EAE rats (h) and C16 late treated EAE rats (i) at week 8 post-immunization. Medium and high dose C16 treatment reduced reactive gliosis (j). ^a^ *P* < 0.05 versus normal rats, ^b^ *P* < 0.05 versus vehicle control rats, and ^c^ *P* < 0.05 versus 0.5 mg/per day C16 treated EAE rats at week 8 after immunization. (k) Late treated C16 treatment also inhibited reactive gliosis to a certain extent. ^a^ *P* < 0.05 versus normal rats; ^b^ *P* < 0.05 versus vehicle control rats; ^c^ *P* < 0.05 versus C16 treated EAE rats at week 2 postimmunization group; ^d^ *P* < 0.05 versus vehicle control rats at week 8 after immunization.

To assess whether C16 treatments could inhibit EAE-induced reactive gliosis at the chronic stage, we examined expression of GFAP, a marker for astrocytes, with Western blot analysis and immunofluorescence label. Westerns blot revealed that the expression of GFAP increased both in spinal cord and cerebral cortex of vehicle control rats (Figure 8). Immunolabeling showed that the astrocytes proliferated and formed visible glial scar (Figures 9(b) and 9(h)). The GFAP expression level and glial scar formations were significantly decreased in both the early and late C16 treated groups (*P* < 0.01, Figure 8(c); *P* < 0.05, Figures 9(j) and 9(k)). However, the medium and high dose C16 treatments, but not the low dose treatment, showed a remarkable inhibition to astrocytes proliferation when compared with the vehicle control (*P* < 0.01, Figures 8 and 9).

### 3.5. C16 Treatment Inhibited Apoptosis and Reduced Neuron Loss in the CNS of EAE Rat Model

**Figure 10:**
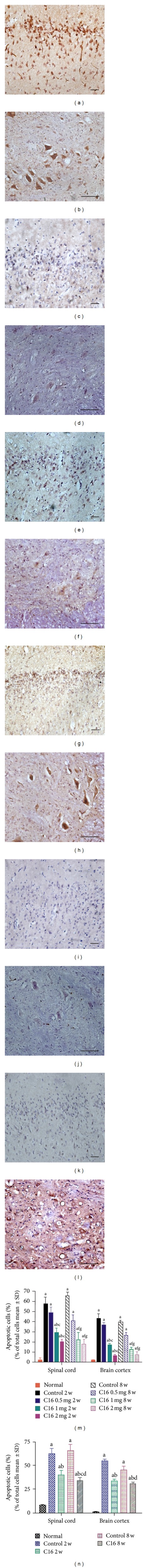
C16 treatment reduced the apoptotic neural cells in spinal cord and brain. Caspase-3 immunostaining, counterstained with hematoxylin; bar = 100 *μ*m. ((a), (c), (e), (g), (i), and (k) coronal sections of motor cortex; (b), (d), (f), (h), (j), and (l) traverse section through the lumbar spinal cord). At week 2 post-immunization, vehicle control rats ((a) large numbers of caspase-3 labeled apoptotic neural cells appeared in the hindlimb area of motor cortex) and ((b) plenty of caspase-3 labeled apoptotic neural cells appeared in the anterior horn of spinal cord) 2 mg/per day C16 treated EAE rats ((c), (d)). C16 late treated EAE rats ((e), (f)). At week 8 post-immunization, vehicle control rats ((g), (h)), 2 mg/per day C16 treated EAE rats ((i), (j)), and C16 late treated EAE rats ((k), (l)). (m) Medium to high-dose C16 treatment reduced the number of caspase-3^+^ apoptotic neural cells. ^a^ *P* < 0.05 versus normal rats; ^b^ *P* < 0.05 versus vehicle control rats at week 2 postimmunization group; ^f^ *P* < 0.05 versus vehicle control rats at week 8 after immunization. ^g^ *P* < 0.05 versus 0.5 mg/per day C16 treated EAE rats at week 8 after immunization. (n) Late treated C16 treatment also decreased caspase-3^+^ apoptotic neural cells to a certain extent. ^a^ *P* < 0.05 versus normal rats; ^b^ *P* < 0.05 versus vehicle control rats at week 2 postimmunization group; ^c^ *P* < 0.05 versus C16 treated EAE rats at week 2 postimmunization group; ^d^ *P* < 0.05 versus vehicle control rats at week 8 after immunization.

**Figure 11:**
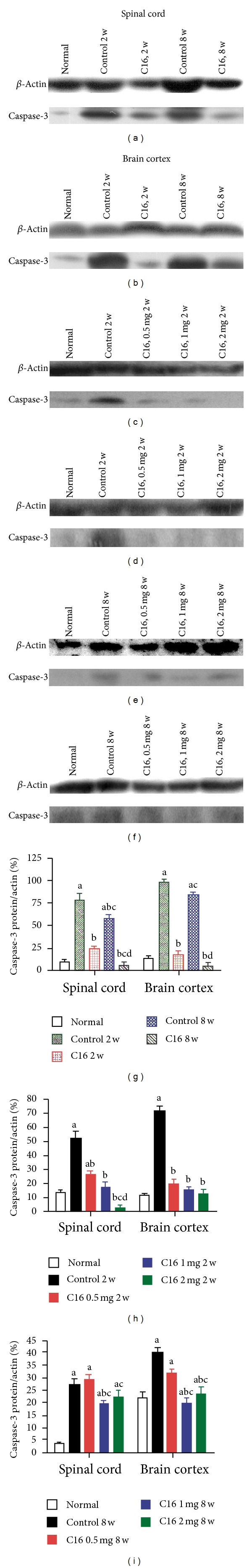
The expression levels of caspase-3 were declined remarkably both in spinal cord (a) and cerebral cortex (b) by late C16 application compared with the vehicle control, evaluated by Western blotting analysis. The levels of caspase-3 expression were evidently decreased both in spinal cord (c) and cerebral cortex (d) by medium to high-dose C16 treatment at week 2 post-immunization. Caspase-3 expression levels were evidently decreased both in spinal cord (e) and cerebral cortex (f) by medium to high-dose C16 treatment at week 8 after immunization. (g) The levels of caspase-3 expression were suppressed by late treated C16 treatment to a certain extent, revealed by Western blotting analysis: ^a^ *P* < 0.05 versus normal rats, ^b^ *P* < 0.05 versus vehicle control rats, ^c^ *P* < 0.05 versus C16 treated EAE rats at week 2 postimmunization group; ^d^ *P* < 0.05 versus vehicle control rats at week 8 after immunization. (h) The levels of caspase-3 expression were reduced by medium to high-dose C16 treatment at week 2 after immunization (i) The levels of caspase-3 expression were reduced by medium to high-dose C16 treatment at week 8 after immunization: ^a^ *P* < 0.05 versus normal rats and ^b^ *P* < 0.05 versus vehicle control rats; ^c^ *P* < 0.05 versus 0.5 mg/per day C16 treated EAE rats, ^d^ *P* < 0.05 versus 1 mg/per day C16 treated EAE rats.

**Figure 12:**
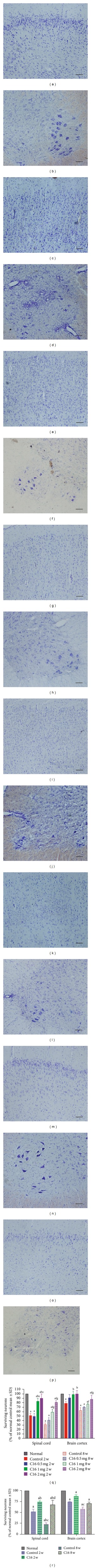
C16 treatment reduced the loss of neurons both in spinal cord and brain. Nissl staining; bar = 100 *μ*m. ((a), (c), (e), (g), (i), (k), (m), and (o) coronal sections of motor cortex; (b), (d), (f), (h), (j), (l), (n), and (p) traverse section through the lumbar spinal cord). At week 2 after immunization, normal rats group ((a), (b)), vehicle control rats ((c), (d)), 2 mg/per day C16 treated EAE rats ((e), (f)), and C16 late treated EAE rats ((g), (h)). At week 8 after immunization, vehicle control rats ((i), (j)), 0.5 mg/per day C16 treated EAE rats ((k), (l)), 2 mg/per day C16 treated EAE rats ((m), (n)), and C16 late treated EAE rats ((o), (p)). (q) C16 treatment increased the surviving neural cells (% of the normal rats) in different dose groups, calculated after Nissl staining. ^a^ *P* < 0.05 versus normal rats; ^b^ *P* < 0.05 versus vehicle control rats at week 2 postimmunization group; ^f^ *P* < 0.05 versus vehicle control rats at week 8 after immunization. ^g^ *P* < 0.05 versus 0.5 mg/per day C16 treated EAE rats at week 8 after immunization. (r) Late treated C16 treatment also lessened the loss of neurons to a certain extent. ^a^ *P* < 0.05 versus normal rats; ^b^ *P* < 0.05 versus vehicle control rats at week 2 postimmunization group; ^c^ *P* < 0.05 versus C16 treated EAE rats at week 2 postimmunization group; ^d^ *P* < 0.05 versus vehicle control rats at week 8 after immunization.

Between week 2 and week 8 aftert immunization, accompanied with severe inflammation, there was a remarkable increase in the number of caspase-3 (an enzyme involved in the execution of the mammalian apoptotic cell death program) immunoreactive neuronal cells in the spinal cords and motor cortexes of vehicle control rats (Figure 10). Western blot analysis also revealed a significant increase of active caspase-3 expression level in vehicle control, which was significantly reversed by C16 treatment, especially in the high dose C16 treated rats (*P* < 0.01, Figure 11). Moreover, the caspase-3 expression level and caspase-3 IR neural cell numbers were also declined evidently in late C16 treated groups from 2 to 8 weeks after immunization (Figure 10). Meanwhile, remarkable neuron loss appeared in the anterior horn of spinal cords and motor cortexes of the vehicle control rats (*P* < 0.01 versus normal control, Figure 12), especially at the late stage of the clinical course (*P* < 0.005 versus normal control, week 8 after immunization). Compared with vehicle control, medium and high dose C16 treatment significantly ameliorated the neuronal loss, both at weeks 2 and 8 after immunization (*P* < 0.01, Figure 12(q)). In the late treated group, significant differences were found between the vehicle and C16 treated rats in the anterior horn of the spinal cord (*P* < 0.05) but not in the motor cortex (*P* > 0.05, Figure 12(r)).

### 3.6. C16 Treatment Suppressed the Expression of TNF-*α* and IFN-*γ* but Showed No Obvious Effect on the Expression of TGF-*β*

**Figure 13:**
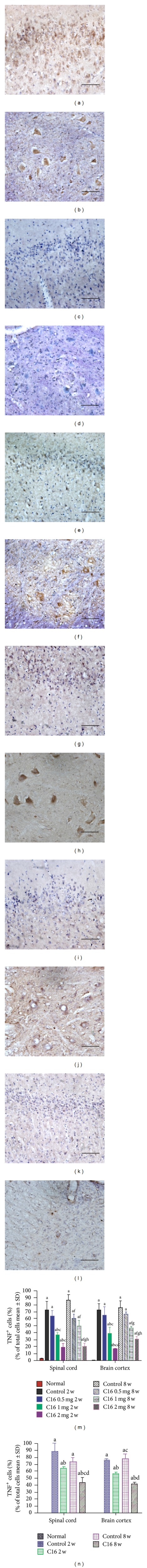
C16 treatment reduced TNF-*α* expression both in spinal cord and brain. TNF-*α* immunostaining, counterstained with hematoxylin; bar = 100 *μ*m. ((a), (c), (e), (g), (i), (k), and (m) Coronal sections of motor cortex; (b), (d), (f), (h), (j), (l), and (n) traverse section through the lumbar spinal cord). At 2 weeks after immunization: vehicle control rats ((a), (b) plenty of TNF-*α* labeled apoptotic neural cells appeared in motor cortex and spinal cord anterior horn), 2 mg/per day C16 treated EAE rats ((c), (d)), and C16 late treated EAE rats ((e), (f)). At week 8 after immunization, vehicle control rats ((g), (h)), 2 mg/per day C16 treated EAE rats ((i), (j)), and C16 late treated EAE rats ((k), (l)). (k) Medium to high-dose C16 treatment reduced TNF-*α* labeled cells at 2 weeks postimmunization group, ^a^ *P* < 0.05 versus normal rats; ^b^ *P* < 0.05 versus vehicle control rats, ^c^ *P* < 0.05 versus 0.5 mg/per day C16 treated EAE rats. At week 8 after immunization, ^f^ *P* < 0.05 versus vehicle control rats, ^g^ *P* < 0.05 versus 0.5 mg/per day C16 treated EAE rats, and ^h^ *P* < 0.05 versus 1 mg/per day C16 treated EAE rats. (l) Late treated C16 treatment also decreased TNF-*α* labeled cells to a certain extent. ^a^ *P* < 0.05 versus normal rats; ^b^ *P* < 0.05 versus vehicle control rats at week 2 after immunization; ^c^ *P* < 0.05 versus C16 treated EAE rats at week 2 after immunization; ^d^ *P* < 0.05 versus vehicle control rats at week 8 after immunization.

**Figure 14:**
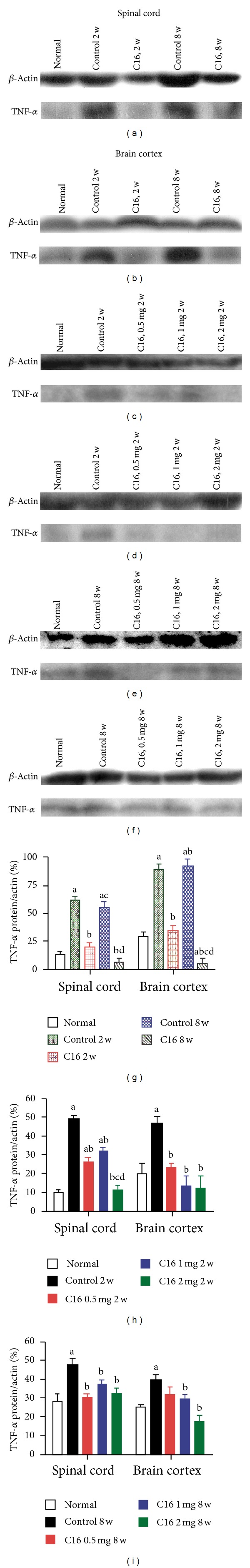
((a), (b)) The levels of TNF-*α* expression were declined remarkably by late C16 application both in spinal cord (a) and cerebral cortex (b) compared with the vehicle control, evaluated by Western blotting analysis. ((c), (d)) The levels of TNF-*α* expression were evidently decreased both in spinal cord (c) and cerebral cortex (d) by medium to high-dose C16 treatment at week 2 post-immunization. ((e), (f)) The levels of TNF-*α* expression were evidently decreased both in spinal cord (e) and cerebral cortex (f) by medium to high-dose C16 treatment at week 8 post-immunization. (g) The levels of TNF-*α* expression were suppressed by C16 late treatment to a certain extent. ^a^ *P* < 0.05 versus normal rats; ^b^ *P* < 0.05 versus vehicle control rats; ^c^ *P* < 0.05 versus C16 treated EAE rats at week 2 postimmunization group; ^d^ *P* < 0.05 versus vehicle control rats at week 8 after immunization. (h) The levels of TNF-*α* expression were reduced by medium to high-dose C16 treatment at week 2 after immunization. (i) The levels of TNF-*α* expression were reduced by medium to high-dose C16 treatment at week 8 after immunization. ^a^ *P* < 0.05 versus normal rats; ^b^ *P* < 0.05 versus vehicle control rats; ^c^ *P* < 0.05 versus 0.5 mg/per day C16 treated EAE rats; ^d^ *P* < 0.05 versus 1 mg/per day C16 treated EAE rats.

At the early (week 2) and late (week 8) stage of the clinical course, extensive expression of TNF-**α**was found in neurons and other neuronal cells in the motor cortex and the spinal cord of vehicle control treated EAE rats (Figures 13(a), 13(b), 13(g), and 13(h)). However, the TNF-*α* IR neuronal cells were much less in C16 treated EAE rats (Figures 13(c)–13(f), and Figures 13(i)–13(l)). The Western blot also demonstrated a remarkable decrease of TNF-*α* expression in each C16 treated group (*P* < 0.01, Figure 14). Such a declining trend could remain from week 2 to 8 after immunization, and was also detected in C16 late treated group (Figures 13(m) and 13(n), Figure 14. *P* < 0.01). 

**Figure 15:**
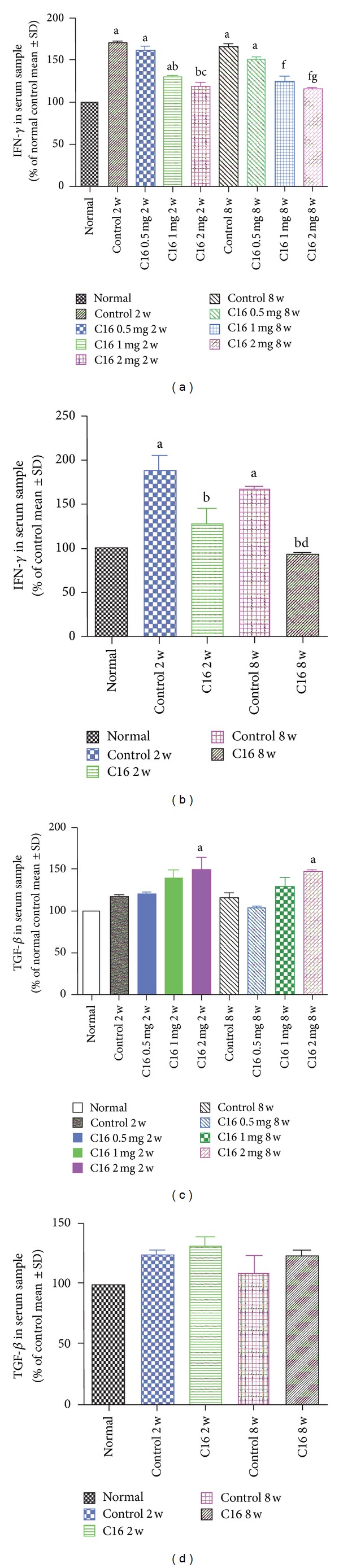
(a) The IFN-*γ* levels in plasma samples measured by ELISA in different dose C16 treated groups. ^a^ *P* < 0.05 versus normal rats, ^b^ *P* < 0.05 versus vehicle control rats, and ^c^ *P* < 0.05 versus 0.5 mg/per day C16 treated EAE rats at 2 weeks after immunization. ^f^ *P* < 0.05 versus vehicle control rats; ^g^ *P* < 0.05 versus 0.5 mg/per day C16 treated EAE rats at 8 weeks after immunization. (a) The IFN-*γ* levels in plasma samples measured by ELISA in C16 late treated group. ^a^ *P* < 0.05 versus normal rats; ^b^ *P* < 0.05 versus vehicle control rats; ^c^ *P* < 0.05 versus 0.5 mg/per day C16 treated EAE rats; ^d^ *P* < 0.05 versus 1 mg/per day C16 treated EAE rats at 8 weeks after immunization. (c) The TGF-*β* levels in plasma samples measured by ELISA in different dose C16 treated group. ^a^ *P* < 0.05 versus normal rats. (d) The TGF-*β* levels in plasma samples measured by ELISA in C16 late treated group.

The expression levels of IFN-*γ* and TGF-*β* in blood serum were measured by ELISA. Results showed that both medium and high doses of C16 application could noticeably reduce the expression of IFN-*γ* and the late treated C16 also possessed similar effects (*P* < 0.05, Figures 15(a) and 15(b)). Nevertheless, as expected, the high dose C16 application induced higher TGF-*β* expression (*P* < 0.05 versus normal rats group) while there was no significantly difference in TGF-*β* expression between low dose C16 treatment and vehicle control rats (*P* > 0.05, Figures 15(c) and 15(d)).

## 4. Discussion

In our study, a peptide that can specifically recognize and bind to *ανβ*3, the integrin that plays an important role in the leukocyte accumulation and adhesion process [14–17], was employed to competitively block transmigration of leukocytes when they were crossing the endothelium. As the antibody to integrin *α*4 has been already reported to prevent leukocyte infiltration in guinea pig EAE models [27], *ανβ*3 integrin inhibition was also discovered to reduce leukocyte-endothelium interaction in a pressure-induced reperfusion model [28]. In agreement with former research [16], our data demonstrated that the widespread perivascular and parenchymal infiltrations of leukocytes, lymphocytes (labeled by pan-leukocyte marker CD45 and lymphocytes marker CD4), and activated microglia and extravasated macrophages (labeled by CD68) in the CNS of rodent EAE model were all significantly suppressed by consecutive intravenous injections of C16. Although we gave daily injection of C16 for 2 weeks, half of the animals survived for 8 weeks. Thus, these sustained neuroprotective effects observed at the later stage of the EAE model (week 8 after immunization) suggested that neurotrophic treatments may have lasting effects, even when treatment has been halted.

In addition to taking part in the infiltration process of inflammatory cells, *ανβ*3 integrin is thought to be an important receptor that regulates macrophage differentiation and macrophage responses to external signaling [29]. Previous studies hypothesize that *ανβ*3 activation can maintain chronic inflammatory processes in pathological conditions [29]; thus, besides interfering with the leukocyte transmigration, blockage of *ανβ*3 may also directly inhibit macrophage-related inflammation.

It is established that MS is not simply an autoimmune disease [30]. In addition to inflammation, the demyelination, axonal injury, and neuronal loss all underlie the accumulation of disability and the disease progression [3]. Extensive demyelination has been confirmed by reduced LFB staining and the loss of MBP immunoreactivity in our vehicle control rats, in which subsequent axonal loss was also prominent in the spinal cords and brain cortexes. The infiltrated inflammatory cells failed to accumulate in C16 treated groups. The inflammatory scores were also clearly decreased compared with that of the vehicle control. Since extravasated inflammatory cells could activate a series of noxious factors that contribute to secondary injury, the improvement of the microenvironment in C16 treated EAE rats should alleviate secondary injury that would otherwise lead to subsequent demyelination, axon loss, and further tissue damage [23]. Therefore, a significant decrease in demyelination areas and relief of axonal damage were detected in C16 treated groups both at week 2 and week 8 after immunization. The less abnormal ultrastructure and the more remyelinated axons under electron microscopy further confirmed the positive effects of C16 on myelin and axons. All these phenomena may be ascribed to the amelioration of the inflammatory milieu. 

The notable increase of the active caspase-3 expression was detected in vehicle treated EAE rats. These phenomena were reversed obviously by C16 treatment. The higher number of intact neurons in the brain cortex and spinal cord anterior horn (showed by Nissel staining and cell counting) of C16 treated EAE rats confers that the improved micro-environment could suppress apoptosis of neural cells. Other than neurons, the oligodendrocytes were also highly vulnerable to an aggravated micro-environment [31]. At chronic stages of neuroinflammation, a large number of oligodendrocytes underwent apoptosis at sites distant from the vicinity of primary injury [31–33], which, led to denudement of axons and deterioration of their conductive abilities, thereby exacerbating the impediment of function [34]. The reduction in neuron and oligodendrocyte apoptosis may contribute to ameliorating disease progression and alleviating disease severity at peak time of EAE, leading to more rapid recovery of locomotor function in C16 treated groups. 

Cytokines produced by the myelin specific CD4^+^ T helper 1 (Th1) cells subset, such as IL-6, IL-12, TNF-*α*, and IFN-*γ*, tend to act in a pro-inflammatory manner, thereby worsening EAE disease process [35]. The proinflammatory cytokines TNF-*α* and IFN-*γ* could further induce a local influx of inflammatory cells into the CNS. Inhibition of these pro-inflammatory cytokines has been shown to be effective in downregulating the EAE disease process [2, 36]. On the other hand, TGF-*β*, the cytokine produced by CD4^+^ T helper 2 (Th2) subset, is anti-inflammatory; it could ameliorate the EAE disease pathogenesis [9]. Our results showed that C16 application could noticeably reduce the expression of TNF-*α* and IFN-*γ* but had no considerable effects on TGF-*β* levels when compared with the vehicle treated EAE rats. Since C16 treatment has been shown to block the accumulation and infiltration of CD4 labeled T cells, pro-inflammatory factors produced by these cells would decrease accordingly, which further inferred the downstream signal transduction pathway and reduced the secondary transmigration of inflammatory cells. On the other hand, the lack of a positive effect on anti-inflammatory cytokine TGF-*β* may be due to similar suppressing effects on Th2 cells subset. Furthermore, the slight increase in TGF-*β* levels over the normal control may, in fact, be a compensation reaction in the inflammatory microenvironments.

Reactive astroglia accumulated within and at the margins of demyelination lesions in MS and EAE, and contributed to the inflammatory response by synthesizing proinflammatory cytokines and presenting peptide antigens to T lymphocytes [11]. A long term result of the astrocytic reaction could be the formation of a glial scar at the lesion site, which may inhibit axonal regeneration or remyelination [12]. With improved regional microenvironment, decreased demyelination, and less pro-inflammatory cytokines, C16 treatment evidently ameliorated reactive astrocytes proliferation, which would be propitious in relieving disability in locomotor function.

Among a variety of EAE models in rodents, the acute EAE model induced in Lewis rats is a well-established model of MS, characterized by a single peak of paralysis after which animals recover spontaneously [37]. The utilization of this model gives us an opportunity to elucidate the induction, peak, and resolution of the inflammation-based immune response of MS. Although animals recover spontaneously in this EAE model, C16 treated groups had less severe clinical score at the same time point in peak and resolution stages when compared with the vehicle control.

Daily intravenous injections of C16 at a higher dose provided significantly better protection to white matter than the low dose, which suggested a dose-dependent effect of C16 application. In another group, the daily injection of C16 started 10 days after immunization, when the symptoms of motor disability would have clinical symptoms, so the case could be diagnosed and intravenous treatments could start. The clinical score was not improved at week 2 after immunization in the C16 late treated group when the C16 injection was only performed for 4 days. However, even in this case, the infiltration of inflammatory cells has been partly reduced and the proinflammatory cytokines/apoptotic signals have also been suppressed. Moreover, a clearly alleviated disease severity and more rapid recovery in locomotor function were detected at week 8 after immunization, after receiving C16 treatment for a 2-week duration. The results implied that delayed C16 treatment could also, at least to a certain extent, offer neuroprotective effects for EAE. The dose and duration of application were important factors in evaluating the effects of C16.

## 5. Conclusion 

Our data suggests that C16 peptide might act as a protective agent by attenuating inflammatory progression, improving micro-environment, and affecting the expression of some proinflammatory cytokines in neuroinflammatory disease. Although we have explored a part of the underlying mechanism of C16 effects, further molecular mechanisms still need extensive investigation. Combination with different innovative factors that target different pathways of neuroprotection should be one more effective route to enhanced therapies. For instance, neurotrophic factors may promote remyelination and prevent neuronal damage; it is possible that they have the potential to be used in a combination of therapeutic drugs that targets neuroprotection pathways other than C16 one. Experiments along these lines are currently in progress in our laboratory.

## Figures and Tables

**Table 1:** Histopathological analysis of demyelination damage in spinal cord (S) and brain cortex (B) of C16 late treated group.

	Control 2 week	C16 2 week	Control 8 week	C16 8 week
Spinal cord	2.20 ± 0.55	1.20 ± 0.42* (*P* = 0.003)	3.40 ± 0.53	1.60 ± 0.54* (*P* = 0.001)
Brain cortex	2.40 ± 0.67	1.40 ± 0.53* (*P* = 0.004)	2.60 ± 0.56	1.80 ± 0.45* (*P* = 0.0035)

Data is presented as mean ± SD. *P < 0.05 was considered statistically significant as compared to control group.

**Table 2:** Histopathological analysis of demyelination damage in spinal cord (S) and brain cortex (B) of C16 dose dependent treated group.

2 weeks	Control 2 w	C16 0.5 mg 2 w (Low dose group)	C16 1 mg 2 w	C16 2 mg 2 w
Spinal cord	3.60 ± 0.57	1.40 ± 0.56* (*P* = 0.0023 versus control*)	1.39 ± 0.50^∗#^ (*P* = 0.001 versus control*; *P* = 0.039 versus low dose group^#^)	0.80 ± 0.45^∗$^ (*P* = 0.00013 versus control*; *P* = 0.006 versus low dose^$^)
Brain cortex	3.20 ± 0.44	1.60 ± 0.55* (*P* = 0.001 versus control*)	1.20 ± 0.45^∗#^ (*P* = 0.001 versus control*; *P* = 0.014 ver*s*us low dose group^#^)	0.60 ± 0.52^∗$^ (*P* = 0.0001 versus control*; *P* = 0.003 versus low dose^$^)

8 weeks	Control 8 w	C16 0.5 mg 8 w	C16 1 mg 8 w	C16 2 mg 8 w

Spinal cord	3.40 ± 0.62	2.8 ± 0.84* (*P* = 0.0052 versus control*)	1.8 ± 0.4^∗#^ (*P* = 0.0021 versus control*; *P* = 0.005 versus low dose group^#^)	1.2 ± 0.45^∗$^ (*P* = 0.0014 versus control*; *P* = 0.0017 versus low dose^$^)
Brain cortex	2.60 ± 0.67	1.4 ± 0.55* (*P* = 0.0018 versus control*)	1.2 ± 0.40^∗#^ (*P* = 0.001 versus control*; *P* = 0.04 versus low dose group^#^)	1.0 ± 0.71^∗$^ (*P* = 0.0009 versus control*; *P* = 0.01 versus low dose^$^)

Data is presented as Mean ± SD. *P < 0.05 was considered statistically significant compared with the control, ^#^ *P* < 0.05 compared with low dose (0.5 mg) C16 treatment group, and ^$^ *P* < 0.05 compared with low dose (0.05 mg) C16 treatment group.

## Abbreviations

EAE: Experimental allergic encephalomyelitis
MS: Multiple sclerosis
CNS: Central nervous system
GFAP: Glial fibrillary acidic protein
ANOVA: Analysis of variance
ABC: Avidin-biotin peroxidase complex
TGF-*β*: Transforming growth factor-beta
TNF-*α*: Tumor necrosis factor-*α*
IFN-*γ*: Interferon-*γ*
ICAM-1: Intercellular adhesion molecule-1.
